# The Impact of Various Tax Bases and Rates on Cannabis Consumption Among US Adults Who use Recreational Cannabis

**DOI:** 10.1002/hec.70129

**Published:** 2026-07-08

**Authors:** Yanyun He, Lei Xu, Hojin Park, Shaoying Ma, Ce Shang

**Affiliations:** ^1^ Center for Tobacco Research The Ohio State University Wexner Medical Center Columbus Ohio USA; ^2^ Department of Economics Chosun University Gwangju South Korea; ^3^ Department of Internal Medicine and Center for Tobacco Research The Ohio State University Wexner Medical Center Columbus Ohio USA

## Abstract

As an increasing number of states legalize recreational cannabis, it is increasingly important to understand the optimal tax design for recreational cannabis. Currently, various tax bases (weight, potency, price) and rates are used. However, the empirical evidence on the impact of tax‐related attributes (pre‐tax prices, tetrahydrocannabinol [THC] levels, tax bases, and tax rates) and their interactions on consumption is limited. To address this evidence gap, this study utilizes a split‐sample volumetric choice experiment to understand the impact of pre‐tax prices, tax bases, tax rates, and THC levels on cannabis consumption and THC intake by US adult users. We further estimated the own‐ and cross‐price elasticities for four cannabis forms (legal flower, illegal flower, edibles, and vaping concentrates). We collected a nationally representative sample of *N* = 1525 US adults aged 21+ who reported the past 30‐day use of recreational cannabis in June and July 2024. Participants were randomized into seeing one of the three different tax bases, and each person answered 9 choice questions where they were given varying combinations of pre‐tax prices, tax rates, and THC levels of the four cannabis products. Zero‐inflated negative binomial regressions were used to estimate the impacts of varying factors on the cannabis consumption (units and THC) and own‐ and cross‐price elasticities. The interactions between tax bases and their targeted attributes were explored using a linear model accounting for individual fixed effects. Higher pre‐tax prices and tax rates significantly reduce consumption, whereas higher THC levels increase consumption. Compared to a tax rate of 60% of pretax prices or equivalent, an 80% rate does not induce further consumption reduction. The overall price elasticities of cannabis demand range from −0.4 to −0.5, with form‐ or product‐specific price elasticities ranging between −0.2 and −0.4. Illegal and legal flowers are substitutes for each other. Edibles and cartridges are complements for each other. In addition, illegal flowers are substitutes for legal cartridges, and edibles are complements to flowers. While increases in the prices of legal products significantly reduce their consumption, 89% of this reduction may be offset by switching to illegal products. While tax bases do not significantly impact consumption, a potency tax base reduces the consumption of higher potency products, and a price tax base reduces the consumption of higher‐priced products. If the illegal market is restricted, policymakers can expect increasing cannabis prices using excise taxes to reduce both unit and THC consumption, while generating tax revenues. However, given the sizable illegal market, a large portion of the consumption reduction due to taxes may be offset by switching to illegal products. Tax rates reduce consumption in addition to base prices. There is room to increase cannabis tax rates to 60% of pretax prices or equivalent to counter the decreasing prices. While tax bases are not effective in reducing overall consumption, they can shift the preference of products with the attributes that they target (e.g., high potency or high price); therefore, they could be a useful tool to shift product choices. Capping THC levels also has a direct impact on reducing consumption.

## 1 Introduction

Cannabis is the most commonly used federally illegal drug by youth and adults in the United States (US) (Substance Abuse and Mental Health Services Administration 2023). In 2021, 18.7% of people aged 12 or older reported cannabis use in the past year, a significant increase from 11% in 2002 (Substance Abuse and Mental Health Services Administration 2020, 2023). Even more concerning is the consistent upward trend in cannabis use prevalence since 2002, with no periods of decline observed (Substance Abuse and Mental Health Services Administration 2020). Despite well‐known therapeutic effects, cannabis use may lead to a wide range of adverse physical and mental outcomes, including worsened respiratory symptoms, lower birth weight of offspring, and development of schizophrenia or other psychoses (National Academies of Sciences Engineering and Medicine et al. 2017). The primary active chemical in cannabis is tetrahydrocannabinol (THC), which is responsible for the psychoactive and euphoric effects and is a primary measure of the potency of the cannabis product.

Strong scientific evidence demonstrates that increasing prices through excise taxation is one of the most effective strategies to reduce cannabis use and its associated harms (Gallet 2014; Mace et al. 2020; Pacula and Lundberg 2013). Economists measure the impact of price changes on consumption using the concept of “price elasticity of demand.” Both global and US studies show that the price elasticity of cannabis demand is −0.1 and −0.8 and is price‐inelastic (i.e., with an absolute value < 1) (Desimone and Farrelly 2003; Gallet 2014; Pacula and Lundberg 2013; Payne et al. 2020; Ruggeri 2013; Aston et al. 2016; Davis et al. 2016; Desimone and Farrelly 2003; Halcoussis et al. 2017; Han et al. 2025; Hansen et al. 2017; Vincent et al. 2017; Xing and Shi 2025). These findings suggest that raising cannabis taxes not only decreases consumption but also increases tax revenue.

The existing evidence on cannabis price elasticity is limited in several ways. First, most studies using observational data or surveys did not address the endogeneity issue of prices, and the only study that used taxes as instruments for prices appears to have limited statistical power (Han et al. 2025). Second, no studies have additionally estimated cross‐price elasticities between different forms and sources of cannabis. Understanding these relationships is important because users may often consume different forms of cannabis together (Leal and Moscrop‐Blake 2024; Schauer et al. 2016; Steigerwald et al. 2018). If two forms are substitutes, higher prices or taxes on one form could unintentionally drive users to increase the consumption of the other. Conversely, if the two forms are complements, price or tax policies targeting one form may also reduce the consumption of the other. To our knowledge, only two experimental studies have examined the relationships between these forms or sources. One study found that legal and illegal cannabis exhibit asymmetric substitution (Amlung et al. 2019), while another observed that flowers and concentrates are weak complements (Panchalingam et al. 2023). However, these experimental studies used hypothetical purchase tasks, which estimate elasticities using a functional form that is not directly comparable to observational data and therefore yield limited values in informing the revenue impacts of taxes and prices. More research is needed to assess own and cross price elasticities by forms and sources.

Given that medical use of cannabis may be beneficial, states usually levy excise taxes only on retail cannabis for recreational use and base these taxes on product characteristics like weight, prices, and potency (Park et al. 2024). Unlike alcohol and cigarette markets, where standard products dominate, it is challenging to choose a common tax base for retail cannabis products due to the variety of product forms and characteristics (Park et al. 2024). According to the literature on tax structures for cigarettes and beer, a weight‐based tax has the advantage of raising prices uniformly, reducing the risk of consumers switching to lower‐priced products (Shang et al. 2019; Shang et al. 2018). However, it may fail to address THC intake, as it does not account for potency. In contrast, a price‐based tax might more effectively reduce THC intake because higher‐potency products are typically more expensive. Yet, it may not create sufficient price barriers to prevent initiation with low‐priced products. Recent evidence also indicates that price differences do not adequately reflect potency differences, making a price‐based tax potentially less effective than a potency‐based tax in reducing THC intake (Hansen et al. 2020). Because THC levels are related to adverse health consequences and have been increasing over time, a potency‐based tax could be a more effective approach than weight‐ or price‐based taxes (Hansen et al. 2020; Smart et al. 2017; Xing and Shi 2025). Despite this, most state tax structures rely on weight‐ and price‐based systems, with Connecticut, Illinois, and New York being the only states to have implemented potency‐based taxation (Hoffer 2023; Park et al. 2024).

The marketplace data further demonstrate that weight, price, and potency, which are commonly used as tax bases for cannabis, are correlated, making the identification of the optimal tax base using observational data challenging (Davenport 2021; Han and Shi 2025; Hansen et al. 2020; Smart et al. 2017). One study used an experimental cannabis marketplace to compare price and potency tax bases and found that cannabis users purchased fewer high‐potency products under a potency tax base than under a price tax base, but the total unit and THC consumption did not differ (Xing and Shi 2025). However, that study did not account for weight‐based taxes, the influence of the illegal market, or the distinction between pre‐tax prices and tax rates. Therefore, the insights generated for taxes based on price and weight, tax rate setting, and how various tax structures (i.e., combinations of bases and rates conditional on prices) influence consumption are limited. More research is needed to comprehensively assess various tax structures and how they influence consumption through the targeted characteristics. Moreover, cannabis excise taxes are based on product characteristics that are constantly evolving in the marketplace, suggesting updated tax rates may be needed in addition to tax base choices. One significant trend is that cannabis potency levels (e.g., THC levels) have been increasing over time (Hansen et al. 2020; Smart et al. 2017). Between 2014 and 2017, the average potency levels increased from 15% to over 20% (Hansen et al. 2020). Another trend is the growing market share of potent concentrates (e.g., cartridges), which have become increasingly popular (Bidwell et al. 2018). In contrast to the increasing potency levels and growing popularity of concentrates in the US, the average price of cannabis products has been decreasing since the retail markets opened (Davenport 2021; Park et al. 2024; Smart et al. 2017). Between 2014 and 2023, cannabis retail prices, wholesale prices, and excise taxes in legal markets, regardless of locations, have all been trending down significantly in both nominal and real terms (Park et al. 2024). Considering these trends, higher tax rates beyond existing levels may be needed to counter decreasing prices and increasing potency levels. However, the research is lacking on the preferred combinations of cannabis tax rates and bases that account for product development and market dynamics.

Finally, the significant and unique challenge when designing tax structures for retail cannabis is the parallel illegal cannabis market (Goodman et al. 2020). Despite the legalization of the retail cannabis market in many states, the illicit market remains an important source of cannabis products (Caulkins et al. 2019). Two reports in 2019 suggest that the illegal market in states with legal sales may be as large as 40%–60% of the total market (Caulkins et al. 2019; Davenport et al. 2019). The existence of illegal products could significantly undermine the effectiveness of taxes. However, almost all existing experiments on cannabis purchases did not explicitly model the influence of illegal markets and simply used price impacts to model tax impacts, which do not reflect reality. Many observational studies also suffer from similar problems, as they use legal market prices and sales to estimate demand.

This study will address these critical knowledge gaps by assessing how tax‐related product attributes (THC levels, pre‐tax prices, tax bases, and tax rates) impact unit and THC consumption, estimating own and cross‐form or cross‐source (legal vs. illegal) elasticities for different cannabis products, and elucidating how tax bases interact with targeting characteristics in influencing consumption. We will use novel volumetric choice experiments (VCEs) that offer several key advantages in methodologies: (1) They enable the estimation of causal effects in a controlled setting by systematically varying key policy variables that cannot be estimated due to correlation and confounding in observational data. For example, by manipulating prices, VCE studies generate exogenous variation, enabling causal interpretation of own‐ and cross‐form elasticity estimates, as well as separating the effects of prices versus taxes and tax bases versus rates; (2) VCE studies offer distinct advantages over real‐world natural experiments by allowing for the pre‐evaluation of tax policies before implementation, such as tax rates beyond current levels to counter decreasing price trends; (3) Compared to other experiments, VCEs offer additional advantages. Compared to discrete choice experiments, VCEs allow for multiple concurrent uses. Compared to hypothetical purchase tasks, VCEs allow for more than two labeled products, and price elasticity estimates from VCEs are more aligned with observational data. Compared to the experimental cannabis marketplace, VCEs are theory‐grounded and allow for systematic variations of more than two attributes while imposing less burden on participants (Adamowicz et al. 1994; McFadden 1972); and (4) They provide insights into products that are under‐reported or not reported in surveys (e.g., illegal products). Results will have critical policy implications that inform how to set tax structures to mitigate harm for states contemplating taxation policies as they legalize and for states and localities with legal retail sales contemplating reforms to their current tax structures.

## 2 Methods

### 2.1 Recruitment and Sampling

This study recruited a nationally representative sample of adults aged 21 or older who use cannabis for recreational purposes in the US through Ipsos Public Affairs (Ipsos) KnowledgePanel in June 2024. Ipsos KnowledgePanel is a probability‐based web panel designed to ensure representativeness of the US adult population. Panel members were recruited using probability selection algorithms for both random‐digit dial (RDD) telephone and address‐based sampling (ABS) methodologies. As such, samples from Knowledge Panel cover all households regardless of their phone status. This probability‐based sampling methodology also improves population coverage, particularly for hard‐to‐reach individuals such as young adults and minority subgroups. Ipsos invited one adult from a representative sample of households to participate in this survey, which consists of questions on real‐world cannabis use behaviors and volumetric choice questions that ask about hypothetical purchases in response to various tax bases and rates.

Eligibility criteria for the study included: (1) being 21 years or older, (2) residing in the US, and (3) reporting past 30‐day use of recreational cannabis. We use these criteria because we would like to focus on recreational users who can legally purchase cannabis in the states with retail sales and, therefore, are more likely to be impacted by the tax structures of recreational cannabis. A total of 1525 participants who met this eligibility criteria answered the survey, which was English‐based and took approximately 20 min to complete. The participants were incentivized through KnowledgePanel's standard sweepstakes entry. Moreover, the data collection process included multiple quality control measures, such as logic checks, response consistency validation, and duplicate prevention, to ensure the reliability of self‐reported cannabis consumption behaviors.

### 2.2 Volumetric Choice Experiment (VCE) Design

#### 2.2.1 Overview of VCE Design

We used a split‐sample VCE design to assess the impact of various tax structures (bases and rates) and tax‐related attributes (pre‐tax price levels and THC levels) on cannabis purchase and consumption among US adult cannabis users. In the split‐sample design, participants were randomly assigned or split into one of the three tax base conditions: weight‐based, price‐based, or potency (THC mg)‐based tax bases. In the next step, participants answered nine hypothetical choice sets under the assigned tax base condition, where pre‐tax prices, tax rates, and THC levels additionally varied to assess their effects on purchasing decisions.

Like discrete choice experiments (DCEs), the split‐sample VCE addresses limitations in observational data such as confounding and correlation. Specifically related to cannabis tax structures, tax bases that states have chosen rarely changed over time and therefore cannot be assessed using observational surveys and causal inference (Park et al. 2024). Moreover, both weight and THC levels are associated with cannabis prices, making it challenging to isolate the impacts of these correlated factors using real‐world data (Smart et al. 2017). Survey data from the real world are known to have limitations in capturing the use of illegal products, and they often ask only about cannabis use status (e.g., a dichotomous answer of yes or no) instead of detailed forms or consumption. These limitations make survey data less suitable to estimate cross‐ and own‐price elasticities of demand by forms or for the intensive margin of consumption that is continuous. Finally, the optimal tax base and rate could be some combination that has not been implemented in the real world, which can be tested in VCEs.

Compared to DCEs and classic VCEs, a split‐sample VCE also has advantages in evaluating cannabis tax structures. DCEs elicit a single choice among multiple labeled products and, as a result, do not allow dual or poly use behaviors. In comparison, VCEs accommodate dual and poly use of multiple cannabis forms or products and better represent market shares. Given that cannabis users commonly use multiple different forms, VCEs are more suitable than DCEs to evaluate cannabis use (Leal and Moscrop‐Blake 2024). Moreover, compared to a classic design without randomization, a split‐sample VCE can build in a randomization condition i.e. not suitable or too complicated to present multiple times, such as tax bases and warning labels. Therefore, a split‐sample VCE is a versatile tool that utilizes both within‐subject and between‐subject variations in identification. In this study, we use the randomization or between‐subject variation to identify the impact of tax base on consumption and the within‐subject variation (i.e., nine choice sets) to identify the impact of other tax‐related attributes (pre‐tax prices, tax rates, and THC levels) on consumption.

#### 2.2.2 Labeled and Opt‐Out Options per Choice Task

In this VCE, we used a labeled design with four THC‐containing cannabis products that were always presented to participants in a choice task (Table 1): flowers from dispensaries (in 1/8 ounce or 3.5 g units), flowers from street vendors (in 1/8 ounce or 3.5 g units), edibles from dispensaries (in units, assorted), cannabis THC cartridges from dispensaries (in units). These unit sizes were chosen based on the observations on weedmaps.com and leafly.com, which show that these sizes are typical units for sale.

**TABLE 1:** Attributes and levels, by cannabis product.

Attributes	Legal Flower (Size = 1/8 ounce pack)	Illegal Flower (Size = 1/8 ounce pack)	Edibles (Size = 1)	Cartridges (Size = 1)
Within‐subject variations (manipulated attributes)				
Pre‐tax price levels (4)	$10, $20, $30, $40	$10, $20, $30, $40	$10, $20, $30, $40	$20, $40, $60, $80
Tax levels	20%, 40%, 60%, 80% of pre‐tax prices or equivalent	0	20%, 40%, 60%, 80% of pre‐tax prices or equivalent	20%, 40%, 60%, 80% of pre‐tax prices or equivalent
THC levels (4) (2 for vaping)	10%, 15%, 25%, 30%	10%, 15%, 25%, 30%	20 , 50, 100, 500 mg	0.5 g, 1g
Between‐subject variations (randomization conditions)				
Tax bases	Weight, price, THC mg	N/A	Weight, price, THC mg	Weight, price, THC mg

*Note:* Pre‐tax prices represent 100%, 200%, and 300% increases from low prices. THC levels represent low, medium low, medium high, and high for products other than cartridges. For cartridges, the two THC levels represent medium‐low and high levels.

We chose the four cannabis products based on market shares and policy importance. According to the existing literature, the three popular forms that take the largest market shares remain flowers (> 50%), concentrates (> 20%), and edibles (> 10%), and consumers report concurrent use of multiple forms (Davenport et al. 2019; Smart et al. 2017). However, based on the nationally representative Behavioral Risk Factor Surveillance System, the most frequently used methods in 2022 are smoking (79.4%), eating (41.6%), and vaping (23%) (Quader et al. 2025). The real‐world purchase questions prior to VCE purchase tasks also confirm these shares by showing that our participants spent 47% on flowers, 28% on edibles, 13% on cartridges, and 12% on all other products combined. Participants could select one or multiple products per choice task or opt not to purchase at all.

#### 2.2.3 Attributes and Levels

As shown in Table 1, we manipulate product attributes at both the between‐subject level (tax base) and the within‐subject level (tax rates, pre‐tax prices, and THC levels). Specifically, we randomly assign participants to see choices under three different tax bases: weight‐based taxes, price‐based taxes, and potency‐based taxes, which generates between‐subject variation that allows us to examine how tax bases impact consumption. We further manipulated three attributes at the within‐subject level, which are pre‐tax prices that do not include any taxes, excise tax rates, and THC levels. In other words, each participant will see various combinations of pre‐tax prices, excise tax rates, and THC levels across choice sets.

We choose the levels of tax rate and base according to existing and potential tax policies. States in the US have adopted weight, price, and THC or potency as tax bases for recreational cannabis. The tax rates reflect a range of levels from 20% to 80% of pre‐tax prices or equivalent. While the low levels of tax rates represent existing tax rates, the high levels represent potential tax rates that may counter the decreasing price trend (Park et al. 2024). The high levels of tax rates also mimic the World Health Organization's recommendation that tax shares in retail prices should be at least 75% for tobacco, which implies an excise tax rate of roughly 80% on a pre‐tax price basis and could be applied to cannabis (World Health Organization). Since tax bases differ, the tax levels under different bases are designed to represent similar total tax dollar amounts across bases.

#### 2.2.4 Choice Set Design

The within‐subject attributes and levels generate 16 profiles (i.e., 4 levels of prices × 4 levels of THC) for illegal flowers, 32 for cartridges, and 64 for legal flowers and edibles. To improve the efficiency of the experiment and reduce respondent fatigue, we employed a D‐efficiency fractional factorial design, which minimizes the generalized variance and maximizes the information obtained about the parameters with the least amount of data (Hensher and Johnson 1981; Louviere et al. 2000; Train 2009). Moreover, our prior study shows that nine choice sets per participant are suitable for VCEs and four versions or blocks are sufficient to cover attribute levels similar to this design, leading to our decision to choose 36 choice sets (4 versions or blocks × 9 choice sets) (S. Ma et al. 2026). Next, Ngene software was used to choose 36 choice sets that balance the design complexity with the coverage of attribute and level combinations (Hensher et al. 2021; Louviere et al. 2008). We further divided these choice sets into four blocks and nine choice sets per block. Participants were randomized into one of the four blocks and completed nine choice sets, with each set containing alternative cannabis products with different price levels, tax rates, and THC levels.

#### 2.2.5 Other Design Features and Validity Checks

Choice experiments are theory‐grounded and widely tested in fields such as marketing, medical decision‐making, health economics, and transportation economics (Bhat 2008; McFadden 1972). Following the rich literature on mitigating hypothetical biases in choice experiments and the best practices to enhance experiment validity, we used cheap‐talk scripts at the beginning of the experiment (see Supporting Information S4: Appendix D) (Haghani et al. 2021a, 2021b). After reading the general instructions on how to complete experiments and the expectation to answer nine choice sets, participants were randomized into seeing a tax base condition, where how taxes increase according to the base was presented (e.g., taxes are higher when potency is higher) using a stimulus (See Supporting Information S2: Appendix Figures B.1, B.2, B.3, and B.4).

In the choice sets, all manipulated attributes (pre‐tax prices, THC levels, and tax bases and rates) are explicitly presented to participants (See Supporting Information S3: Appendix C). After assuming that taxes are fully passed to prices, the final price of each product was also calculated based on the pre‐tax price, tax rate, and the respective taxation base (weight, price, or potency) and presented to aid the decision‐making processes.

A budget reminder is also recommended in the literature to improve experiment validity. Prior to the experiment, we asked participants to report their monthly or weekly budget spent on cannabis and to make purchases for the next month, considering their monthly budget. While participants were allowed to go beyond or below this personalized budget, the total spending in dollars of their hypothetical purchases was calculated and compared to their monthly spending in the real world, adding a layer of reality. The instructions that include a cheap‐talk script are presented in Supporting Information S4: Appendix D and the budget reminder and choice set display examples are available in Supporting Information S3: Appendix C.

### 2.3 Measures

#### 2.3.1 Outcomes

The outcome variables were the units or quantities of cannabis and associated THC consumption in mg, as reported in the choice sets in response to attributes and for monthly use. The budget constraints are also set on the monthly budget. The detailed distribution of the cannabis unit consumption and THC consumption levels is shown in Supporting Information S1: Appendix A (Figure A.1 & A.2). Quantity consumption was measured based on popular or common units (see 2.2.2. Labeled and opt‐out options per choice task). THC consumption was calculated by multiplying the number of units chosen by the associated THC levels and was measured in mg. Both unit and THC consumption measures are integral values.

#### 2.3.2 Explanatory and Control Variables

The main explanatory variables are manipulated attributes and levels presented in Table 1, including pre‐tax prices, tax bases and rates, and THC levels. We also calculate tax‐inclusive final prices to estimate own and cross price elasticities. The control variables include monthly budget constraint, alternative‐specific constants for each product, and participants' demographic and socioeconomic characteristics, such as age, sex, race/ethnicity, highest education, and annual household income. Since we have estimated different model specifications, the explanatory and control variables may vary, with details described in the empirical strategy.

## 3 Empirical Strategy

### 3.1 Analytical Sample

The analytical sample size for VCEs is the product of the number of participants, choice sets, and the number of products per choice set. Therefore, with a sample of 1525 participants, 9 choice sets per participant, and four products per choice set, we have a total sample size of 54,900. After dropping 1420 observations due to skipping choice sets, we reached a final analytical sample size of 53,480.

### 3.2 Model to Assess the Impact of Tax‐Related Attributes on Consumption

In the first model (Specification 1), we examine how the manipulated attributes, which are tax bases, tax rates, pre‐tax prices, and THC levels, impact cannabis unit and THC consumption. This specification not only informs the impact of various combinations of tax bases and rates on consumption, but also tests the hypotheses that the impacts of prices and taxes on consumption may be non‐linear (Amlung et al. 2019; Hoffer 2023).

(1) $$\begin{array}{ll} Consumption_{\mathrm{ist}} & =\alpha_{0}\;+\;\alpha_{1}\;ASC_{\mathrm{ist}}+\;\alpha_{2}\;PriceLevel_{\mathrm{ist}}\;+\;\alpha_{3}\;THCLevel_{\mathrm{ist}}\;\\ & \;+\;\alpha_{4}\;TaxRateLevel_{\mathrm{ist}}\;+\;\alpha_{5}\;TaxBase_{i}\;+\;\alpha_{6}\;X_{i}\;+\;\lambda_{m}\;+\;\varepsilon_{\mathrm{ist}} \end{array}$$

In this equation, “i” denotes participants, “s” denotes products, and “t” denotes choice sets. Given the nature of the VCE data, any labeled product not purchased by a participant contributes a zero consumption, resulting in a large number of zeros in the outcome variables (75% of outcomes are zeros). Following the literature, we use a zero‐inflated negative binomial (ZINB) regression to model the impacts of manipulated attributes on consumption (Carson et al. 2022; Chalak et al. 2023; Do et al. 2025; S. Y. Ma et al. 2025). In addition, both unit and THC consumption have a variance greater than the mean, which indicates overdispersion. Therefore, ZINB is a better modeling choice than the zero‐inflated Poisson model. Nonetheless, we also conduct additional checks by treating outcomes as a continuous variable and estimating a two‐part model. This approach involves using logit regressions to estimate the impacts of attributes on whether to purchase and then using OLS regressions to estimate the impacts of attributes on positive consumption. To understand whether there is heterogeneity in product preference, we also conducted subgroup analysis by whether users use cannabis daily or not and by age stratification (21–40 vs. older than 40).

The attribute variables are dummy coded based on levels, which are pretax price levels ($PriceLevel_{i}st$, with $10 or $20 as omitted category, 100% increase, 200% increase, 300% increase), THC levels ($THCLevel_{i}st$, with low as omitted category, medium low, medium high, and high; and only low vs. high levels for cartridges), tax rates ($TaxRateLevel_{i}st$, with 20% of price or equivalent, 40%, 60%, and 80%), and tax bases ($TaxBase_{i}$, with weight based as omitted category, price based, THC based).

The control variables include alternative‐specific constants $\left(ASC_{i}st\right)$ for each labeled product (legal flower, illegal flower, edible, and cartridge), individual‐level variables $X_{i}$, including cannabis monthly expenditures, age in years, sex (male as reference, female, not reported), race/ethnicity (White, Non‐Hispanic as references; Black, Non‐Hispanic; Other, Non‐Hispanic; two or more races, Non‐Hispanic; Hispanic), education (no high school diploma or GED as reference, high school graduate, some college, Bachelor's degree or higher), and annual household income (<$10,000 as reference, $10,000‐$24,999; $25,000‐$49,999; $50,000‐$74,999; $75,000‐$99,999; $100,000‐$149,999; $150,000 or more), and state (*m*) fixed effects (λ_m). We also conducted a sensitivity analysis without controlling for state fixed effects. We cannot control for individual fixed effects for two reasons. First, individual fixed effects are perfectly collinear with the between‐subject attribute—tax bases or the randomization condition. Second, ZINB is a maximum likelihood estimation approach, which is not equipped to estimate 1525—an extremely large number of individual fixed effects without leading to problems such as nonconvergence or the incidental parameters problem.

For dummy‐coded categorical explanatory variables, we report marginal effects estimated using Stata's command “eydx”, which are semi‐elasticities estimating the percent change in the outcomes in response to a one‐unit change from the reference group of the explanatory variable. The standard errors are clustered at the individual level to adjust for repeated observations (i.e., purchases) from same individuals.

### 3.3 Model to Estimate the Own‐ and Cross‐Price Elasticities

In the second model (Equation 2), we estimate own and cross price elasticities among legal flowers, illegal flowers, edibles, and cartridges. These elasticities will inform the inter‐relationship between products or forms, such as whether they are complements or substitutes. These estimates also provide information on whether increasing taxes will drive consumers to illegal products and whether higher tax rates on a certain form (e.g., cartridges and edibles in Illinois) will drive the consumption to a lower‐taxed form (e.g., flowers in Illinois). In Equation (2), most variables are previously defined, except for products' own final tax‐inclusive prices $\left(OwnPrice_{\mathrm{ist}}\right)$ and the corresponding prices of other products or alternatives $\left(OtherPrice_{i\left(j!=s\right)t}\right)$ in the same choice set. Price elasticities are estimated using Stata's command “eyex”, which indicate the percent change in the outcome in response to a one‐percent increase in prices.

(2) $$\begin{array}{ll} Consumption_{\mathrm{ist}} & =\beta_{0}\;+\;\beta_{1}\;ASC_{\mathrm{ist}}\;+\;\beta_{2}\;OwnPrice_{\mathrm{ist}}\;+\;\beta_{3}\;OtherPrice_{i\left(j!=s\right)t}\;\\ & \;+\;\beta_{4}\;THCLevel_{\mathrm{ist}}\;+\;\beta_{5}\;TaxBase_{i}\;+\;\beta_{6}\;X_{i}\;+\;\lambda_{m}\;+\;\varepsilon_{\mathrm{ist}} \end{array}$$

### 3.4 Model to Adjust for Individual Fixed Effects and Assess the Interactions Between Tax Bases and Other Attributes

In the third model (Equation 3), we explore alternative specifications where we control for individual fixed effects and fully rely on within‐subject variation in the attributes to identify their causal impacts on consumption. To do so, we log‐transformed the outcome using log (outcome+1) into continuous variables and used an Ordinary Least Squares linear fixed effects model to control for individual fixed effects. Although we cannot estimate the main effects of tax bases because they are only manipulated between subjects, we are able to estimate the interaction terms between tax bases and other tax‐related attributes, including pre‐tax prices, tax rates, and THC levels. In Model 3 (Equation 3), $Price_{\mathrm{ist}}$, $Tax_{\mathrm{ist}}$, and $THC_{\mathrm{ist}}$ are ordinal variables for attribute levels, respectively. $I_{i}$ denotes individual fixed effects. All other right‐hand variables are previously defined.

(3) $$\begin{array}{ll} Consumption_{\mathrm{ist}} & =C_{0}\;+\;C_{1}\;ASC_{\mathrm{ist}}\;+\;C_{2}\;Price_{\mathrm{ist}}\;+\;C_{3}\;Tax_{\mathrm{ist}}\;+\;C_{4}\;THC_{\mathrm{ist}}\;\\ & \;+\;C_{5}\;\left(Price_{\mathrm{ist}}\times TaxBase_{i}\right)\;+\;C_{6}\;\left(Tax_{\mathrm{ist}}\times TaxBase_{i}\right)\;\\ & \;+\;C_{7}\;\left(THC_{\mathrm{ist}}\times TaxBase_{i}\right)\;+\;I_{i}\;+\varepsilon_{\mathrm{ist}} \end{array}$$

## 4 Results

A summary of the sample characteristics is presented in Table A.1. The average age of our adult recreational cannabis users (aged 21 or above) is 49. The sex distribution is 46% females and 54% males. 73% of the participants are White, Non‐Hispanic. 42% of participants have a bachelor's degree or higher. 37% have an annual household income over $100,000. The average monthly spending on cannabis is $118, with the spending shares being 47% on flowers, 28% on edibles, 13% on cartridges, and 12% on all other products combined. Among these cannabis users who consumed cannabis in the past 30 days, 54% used cannabis daily and 46% used cannabis nondaily. 54.3% of participants claimed that 100% of their real‐world purchases are from legal sources.

Table 2 presents the estimated semi‐elasticities of how pre‐tax price levels, THC levels, tax bases, and tax rates on adult users' cannabis consumption (Model 1), measured in terms of unit and THC consumption (Columns 2 and 4 are preferred specifications with state fixed effects). We find that increases in pre‐tax prices and tax rates are significantly associated with a substantial reduction in both unit and THC consumption, consistent with economic principles of price elasticity. Specifically, compared with the base price, 100%, 200%, and 300% increases in pretax prices result in 53%–54%, 93%, and 121% reduction in unit and THC consumption, respectively. These estimates imply a price elasticity of demand ranging from −0.4 to −0.5.

**TABLE 2:** The impact of attributes on cannabis consumption, estimated using zero‐inflated negative binomial regressions, *N* = 53,480.

	(1)	(2)	(3)	(4)
Variables	Outcome = unit		Outcome = THC	
Within‐subject effects:				
Pre‐tax price levels (base: $10 for flowers and edibles; $ 20 for cartridges)				
100% increase	−0.522***	−0.527***	−0.540***	−0.543***
	(0.036)	(0.027)	(0.034)	(0.028)
200% increase	−0.909***	−0.934***	−0.902***	−0.926***
	(0.043)	(0.033)	(0.044)	(0.034)
300% increase	−1.169***	−1.208***	−1.170***	−1.210***
	(0.058)	(0.048)	(0.056)	(0.048)
THC levels (base: Low)				
Medium low	0.148**	0.128**	0.734***	0.721***
	(0.056)	(0.045)	(0.062)	(0.047)
Medium high	0.241***	0.245***	1.337***	1.341***
	(0.050)	(0.042)	(0.053)	(0.042)
High	0.230***	0.218***	1.982***	1.968***
	(0.053)	(0.044)	(0.056)	(0.046)
Tax levels (base: 20% or equivalent)				
40% or equivalent	−0.149*	−0.141**	−0.209***	−0.209***
	(0.060)	(0.050)	(0.058)	(0.048)
60% or equivalent	−0.292***	−0.289***	−0.312***	−0.318***
	(0.043)	(0.040)	(0.037)	(0.034)
80% or equivalent	−0.298***	−0.323****	−0.347***	−0.371***
	(0.051)	(0.043)	(0.045)	(0.038)
Between‐subject Effects:				
Bases of Tax imposed (base: Weight)				
Price	0.127	0.080	0.186*	0.119
	(0.093)	(0.070)	(0.088)	(0.066)
THC	0.026	0.003	0.073	0.040
	(0.089)	(0.073)	(0.084)	(0.071)
State fixed effects	No	Yes	No	Yes

*Note:* Marginal effects or semi‐elasticities estimated using eydx are reported. Standard errors clustered at the individual levels are in parentheses. For all within‐subject effects, the impacts of levels are significantly different from each other with a *p*‐value < 0.05, except for tax rate levels of 60% and 80% of prices or equivalent.

^*^ *p* < 0.05.

^**^ *p* < 0.01.

^***^ *p* < 0.001.

The findings also reveal that higher THC levels increase consumption. Compared to the low potency level (e.g., 10% for flower products), medium low to high THC levels increase unit consumption by 13%–25% and THC consumption by 72%–197%, respectively. Higher tax rates reduce legal flower consumption when illegal flowers are provided as an option. Compared to taxes at 20% of prices or equivalent, those at 40%, 60%, and 80% of prices or equivalent lead to 14%, 29%, and 32% reduction in unit consumption, respectively. These increases in tax rates also reduce THC consumption by 21%, 32%, and 37%, respectively. The findings suggest a cannabis tax elasticity of demand ranging from −0.1 to −0.2. Additional tests further illustrate that raising tax rates beyond 60% of prices or equivalent to 80% or equivalent does not lead to a significant additional reduction in consumption. Tax bases do not have a significant impact on cannabis consumption among adult recreational users. The regression results for the remaining control variables are presented in Supporting Information S1: Appendix Table A.2.

Sensitivity checks using a two‐part model yield similar conclusions (results by request). Subgroup analyses (Supporting Information S1: Appendix Table A.4) by whether users use daily and age groups suggest very similar results by these stratifications, suggesting limited heterogeneity in experimental responses.

Table 3 presents the own‐ and cross‐price elasticities of demand for four cannabis product forms: legal flower, illegal flower, edibles, and concentrates, estimated using ZINB (Model 2). The own‐price elasticity estimates indicate that all cannabis forms are price‐sensitive. A 10% increase in prices reduces the unit and THC consumption of legal flowers, illegal flowers, edibles, and cartridges by 3%, 3%, 2%, and 4%, respectively. These elasticities are generally not different from one another except for cartridges, which have a greater own price elasticity than other forms. This is likely due to the fact that cartridges tend to contain high THC concentrates and are priced higher per unit than other forms (Smart et al. 2017).

**TABLE 3:** Own‐ and cross‐price elasticities of demand among different cannabis forms, estimated using zero‐inflated negative binomial regressions, *N* = 53,480.

	(1)	(2)	(3)	(4)
	Outcome = unit		Outcome = THC	
Within‐subject effects:				
Own‐price elasticity				
Legal flower	−0.271***	−0.269***	−0.266***	−0.292***
	(0.028)	(0.035)	(0.030)	(0.022)
Illegal flower	−0.270***	−0.267***	−0.265***	−0.271***
	(0.013)	(0.020)	(0.013)	(0.012)
Edible	−0.240***	−0.245***	−0.232***	−0.233***
	(0.021)	(0.022)	(0.022)	(0.020)
Cartridge	−0.412***	−0.428***	−0.431***	−0.413***
	(0.039)	(0.057)	(0.039)	(0.032)
Cross‐price elasticity				
Legal flower				
Illegal flower price	0.043**	0.048**	0.042**	0.049***
	(0.013)	(0.017)	(0.014)	(0.013)
Edible price	−0.006	−0.002	−0.018	−0.007
	(0.018)	(0.019)	(0.018)	(0.013)
Cartridge price	−0.000	0.010	−0.012	0.009
	(0.022)	(0.021)	(0.023)	(0.017)
Illegal flower				
Legal flower price	0.073***	0.085***	0.128***	0.136***
	(0.020)	(0.025)	(0.019)	(0.015)
Edible price	−0.003	−0.001	−0.012	−0.004
	(0.013)	(0.013)	(0.013)	(0.010)
Cartridge price	−0.010	0.015	0.036*	0.043**
	(0.017)	(0.021)	(0.018)	(0.016)
Edible				
Legal flower price	0.045**	0.047**	−0.064***	−0.071***
	(0.016)	(0.017)	(0.017)	(0.016)
Illegal flower price	0.052***	0.057***	−0.034**	−0.034**
	(0.010)	(0.012)	(0.011)	(0.010)
Cartridge price	0.015	0.004	−0.083***	−0.086***
	(0.018)	(0.023)	(0.020)	(0.018)
Cartridge				
Legal flower price	0.012	−0.010	0.019	0.023
	(0.024)	(0.028)	(0.025)	(0.023)
Illegal flower price	−0.003	−0.043	−0.012	0.007
	(0.020)	(0.033)	(0.024)	(0.016)
Edible price	−0.041	−0.059*	−0.064**	−0.047*
	(0.022)	(0.025)	(0.022)	(0.020)
State fixed effects	No	Yes	No	Yes

*Note:* Price elasticities estimated using eyex are reported. Standard errors clustered at the individual level in parentheses.

^*^ *p* < 0.05.

^**^ *p* < 0.01.

^***^ *p* < 0.001.

The cross‐price elasticities reveal important substitution and complementary effects among different cannabis product forms. A summary of the relationship table based on THC consumption is in Supporting Information S1: Appendix A (Table A.5). Legal and illegal flowers are substitutes for each other, with a 10% increase in illegal flower prices leading to a 0.5% increase in legal flower consumption (units and THC). Conversely, a 10% increase in legal flower prices results in a 0.9%–1% increase in illegal flower consumption (units and THC). While the cross‐price elasticities for unit demand between legal and illegal flowers are symmetric (not statistically significant), the cross elasticities for THC demand suggest that the increase in THC from illegal flowers in response to higher legal flower prices exceeds the increase from legal flowers in response to higher illegal flower prices.

Illegal flowers are also substitutes for cartridges. When cartridge prices increase by 10%, the THC consumption from illegal flowers increases by 0.4%. Edibles' relationship with other forms depends on consumption measures. In terms of unit consumption, edibles are likely substitutes for legal and illegal flowers, with a 10% increase in flower prices leading to a 0.5%–0.6% increase in edible unit purchases. However, when looking into THC consumption, edibles are complements to other forms, with a 10% increase in prices of other forms leading to a 0.3%–0.9% decrease in edible THC consumption. Cartridges are also complements to edibles, suggesting their relationship with edibles is symmetric. If edible prices increase by 10%, the unit and THC consumption from cartridges reduces by 0.5%–0.6%. The regression results for the remaining control variables are presented in Supporting Information S1: Appendix Table A.3.

Table 4 presents the results of how within‐subject attributes and their interactions impact consumption, estimated using an OLS model with individual fixed effects (Model 3). Consistent with findings in Models 1 and 2, higher pre‐tax prices and tax rates decrease consumption, and higher THC increases consumption. The interaction terms further demonstrate that, compared to other bases, a potency tax base reduces the effects of THCs on increasing or boosting consumption. This suggests the effectiveness of a potency tax base in reducing consumers' preference for high potency products. In addition, compared to other tax bases, a price tax base significantly amplifies the impact of pre‐tax prices on reducing consumption. The combined findings suggest that tax bases significantly alter consumption and preference for products through the characteristics that they target (i.e., potency taxes impact consumption through potency preferences, price‐based taxes impact consumption, and enhance price effects).

**TABLE 4:** Effects of product attributes, pricing, and taxation on cannabis consumption, *N* = 53,480.

	(1)	(2)	(3)	(4)
	Outcome = unit		Outcome = THC	
THC	0.024***	0.026***	0.269***	0.275***
	(0.003)	(0.005)	(0.013)	(0.022)
THC × price‐based tax		0.011		0.056
		(0.006)		(0.030)
THC × potency‐based tax		−0.020**		−0.075*
		(0.007)		(0.031)
Price	−0.084***	−0.075***	−0.355***	−0.334***
	(0.003)	(0.004)	(0.013)	(0.020)
Price × price‐based tax		−0.020**		−0.083**
		(0.007)		(0.031)
Price × potency‐based tax		−0.006		0.021
		(0.007)		(0.030)
Tax	−0.017***	−0.015*	−0.085***	−0.075**
	(0.002)	(0.006)	(0.009)	(0.029)
Tax × price‐based tax		−0.001		−0.014
		(0.010)		(0.045)
Tax × potency‐based tax		−0.006		−0.016
		(0.011)		(0.047)
Constant	0.489***	0.489***	2.195***	2.195***
	(0.014)	(0.014)	(0.070)	(0.070)

*Note:* Semi‐elasticities are reported. Standard errors clustered at the individual level in parentheses.

^*^ *p* < 0.05.

^**^ *p* < 0.01.

^***^ *p* < 0.001.

## 5 Discussions

In this study, we designed a VCE using a theory‐grounded approach to investigate how tax‐related product attributes, including pre‐tax prices, THC levels, tax rates, and tax bases, influence both cannabis and THC consumption, further exploring the interactions between tax bases and their targeted product attributes. Our findings indicate that higher prices and taxes are associated with lower cannabis consumption and THC intake. The overall price elasticities of cannabis demand range from −0.4 to −0.5, with form‐ or product‐specific price elasticities ranging between −0.2 and −0.4. These findings are consistent with previous studies in showing that cannabis demand is price‐inelastic, with the elasticity estimates falling within the established range of −0.1 to −0.79 (Clements and Daryal 1999; Daryal 2002; Davis et al. 2016). Therefore, if the impacts of illegal markets are limited, policymakers can expect increasing cannabis prices using excise taxes to reduce both unit and THC consumption, while generating tax revenues.

A unique contribution of our study is its findings in cross‐form or cross‐product elasticities considering multiple forms and sources in terms of legal versus illegal flowers. Consistent with Amlung et al. (2019), legal and illegal flowers are substitutes, and THC intake from illegal flower respond more to legal flower prices than the response of THC intake from legal flowers to illegal flower prices (Amlung et al. 2019). However, based on our design, the interpretation of our results is different, as the findings mean that increasing prices through taxes on legal products will drive consumption to illegal products. In addition, illegal flowers are substitutes for legal cartridges. Edibles are complements to flowers and cartridges. Cartridges are complements to edibles. These findings are likely driven by similarities in products, as legal and illegal flowers are more similar than they are to other forms and therefore more substitutable. In contrast, edibles are digested or orally taken, unlike cartridges and flowers, which are often inhaled via either vaping or smoking. Therefore, edibles might act as complements to other products.

Using the reported THC consumption by forms in VCEs and price elasticity estimates (Supporting Information S1: Appendix A.1, 740 mg for legal flowers, 1649 mg for illegal flowers, 105 mg for edibles, and 177 mg for concentrates; price elasticities from Column 4 of Table 2), a 10% increases in legal prices will result in a reduction of 33.5 mg THC consumption from legal products and an increase of 29.7 mg in illegal flower purchases. As a result, there will still be net effects of increasing prices on reducing THC consumption, just that 89% of the estimated reduction may be offset by switching to illegal products. Our conclusions are also supported by existing evidence that, if not considering sources, the price elasticity of cannabis demand is usually inelastic. However, when limiting data to legal sales, price elasticities are large and elastic, suggesting that the availability of illegal sources could make legal sales more responsive to prices (Dodd et al. 2025).

The cross‐elasticities further demonstrate that tiered rates that impose higher taxes on cannabis edibles and cartridges than on flowers, such as those in Illinois, may lead to desirable reductions as edibles and cartridges are price‐responsive and are complements to each other, meaning that the tax impacts will be amplified by cross‐elasticities. Moreover, price increases in edibles and cartridges do not drive users to legal flowers, leading to a possible overall reduction in THC consumption. However, with the existence of illegal flowers, some cartridge users may switch to illegal flowers.

We also estimated how various tax‐related attributes jointly impact unit and THC consumption, which could explicitly inform tax and other cannabis policies. First, we found that tax rates have additional impacts on consumption on top of base prices. Second, tax rates could be set between 20% and 60% of prices or equivalent to counter price decreases and reduce consumption. Considering that the average excise tax incidence among retail prices was about 17% with a range of 8%–50% between 2014 and 2023, most states should have room to increase tax rates (Park et al. 2024). However, tax rates at 80% of prices or equivalent do not provide additional reductions in consumption compared to a 60% rate. THC levels increase consumption due to consumers' preference for higher THC products. Therefore, capping THC levels could reduce consumption.

The findings related to tax bases suggest that while tax bases do not significantly impact the overall unit or THC consumption, they shift preference based on targeting characteristics. Specifically, a THC tax base reduces preference or consumption of higher THC products, and a price tax base further reduces the consumption of higher‐priced products and thereby enhancing the impact of prices on these products. The findings pertaining to potency tax basis are consistent with Xing and Shi (2025) and further illustrate that both potency and price‐based taxes function through the targeted product attributes that are used as bases (Xing and Shi 2025). Both studies also find no significant impacts of tax bases on overall unit and THC consumptions, suggesting tax bases mostly shift the choices of products or their distributions. Therefore, if high potency or high‐priced products (e.g., cartridges vs. others) are considered more harmful due to potency levels or due to their more problematic administration methods (e.g., smoking, vaping, or digestive), tax bases could be a useful policy tool. However, to reduce over THC or unit consumption, higher prices or taxes are effective, but tax bases are not.

Our results further demonstrate the validity of cannabis VCE after comparing the estimates of market shares and price (tax) elasticities with observational data—a common approach to evaluate or mitigate hypothetical biases (Pacula and Lundberg 2013; Payne et al. 2020). We estimated a range of price elasticities closely aligned with what observational data suggest in the literature. The consumption by forms in VCEs reflects the ranking of how participants spent on these forms in real life, and the proportions of purchase are reasonably similar to what the Behavioral Risk Factor Surveillance System (BRFSS) data report (Quader et al. 2025). Moreover, although higher proportions of illegal purchases are expected due to the inclusion of tax rates higher than those in real life, the VCE choices generally show that the illegal purchases account for 55% of unit purchases and 62% of THC purchases. These estimates are not only close to their real‐life behaviors (46% of participants purchased illegal products) but also consistent with prior research suggesting that the illicit market still accounts for 40%–60% of total cannabis sales (Caulkins et al. 2019; Davenport et al. 2019). The above comparisons provide support for the VCE's validity in reflecting real‐world behaviors.

This study has some limitations. First, although our data were weighted to reflect a nationally representative sample of adult recreational cannabis users, we did not apply these weights in our analyses due to the complexity of VCE data and modeling. Therefore, our results may not be generalizable to US users of cannabis, especially those who use cannabis for medical purposes. Second, we did not explicitly model tax avoidance other than tax evasion by buying illegal flowers. In real life, consumers may either buy cheaper products from other states or have the taxes waived if they have medical cannabis certificates. Third, we assume that taxes are fully passed to prices and presented as taxes. However, cannabis taxes in the real market could be either under‐ or over‐shifted to prices. Finally, while our VCE results are generally aligned with real‐world data, future research can combine the VCE results with observational data for further calibration, which may improve the precision of the policy prediction. With these limitations in mind, we do believe that the findings add new information to the literature and cannabis tax policymaking, with several policy recommendations that are important to consider.

## Funding Statement

This study was supported by the National Institutes of Health (NIH)/National Institute on Drug Abuse (NIDA) (grant number: R01DA053294). The content is solely the responsibility of the authors and does not necessarily represent the official views of the NIH/NIDA.

## Conflicts of Interest

The authors declare no conflicts of interest.

## Supporting information


Supporting Information S1

Supporting Information S2

Supporting Information S3

Supporting Information S4

## Data Availability

The data that support the findings of this study are available from the corresponding author upon reasonable request.
