# Supplementary material for: The Impact of Various Tax Bases and Rates on Cannabis Consumption Among US Adults Who use Recreational Cannabis
Source: Health Econ. 2026 Jul 8;35(10):1519–31. doi: 10.1002/hec.70129 (PMC13508039; doi:10.1002/hec.70129)
Supplement: Supplementary file 1 [file HEC-35-1519-s002.pdf]

Appendix B. VCE Survey Stimuli Examples

Figure B.1 Example of Weight-Based Cannabis Tax Stimuli for Flower Products

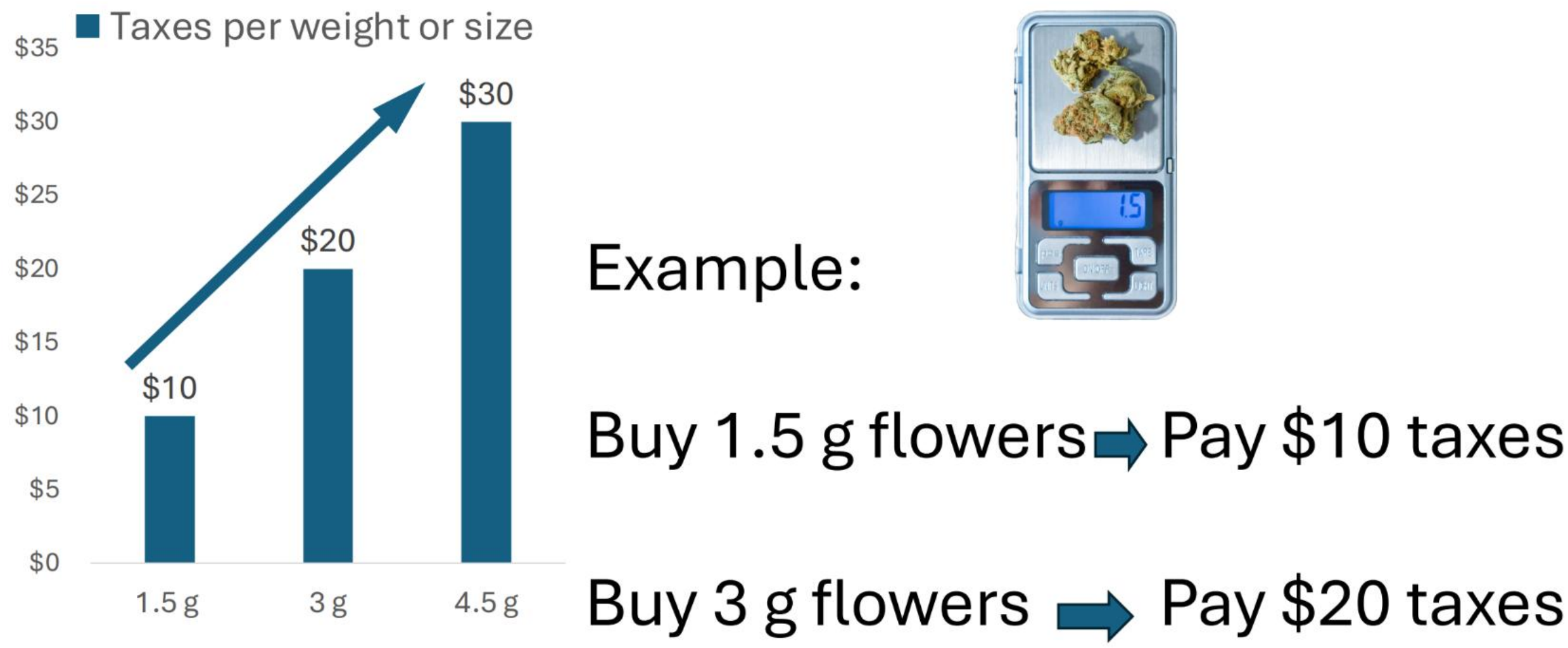

Notes: The figure illustrates the design of the weight-based tax condition, where taxes increase proportionally with the package weight (e.g., $10 for 1.5 g, $20 for 3 g, and $30 for 4.5 g). The example on the right shows a 1.5 g flower purchase corresponding to a $10 tax payment.

Figure B.2 Example of THC-Based Cannabis Tax Stimuli

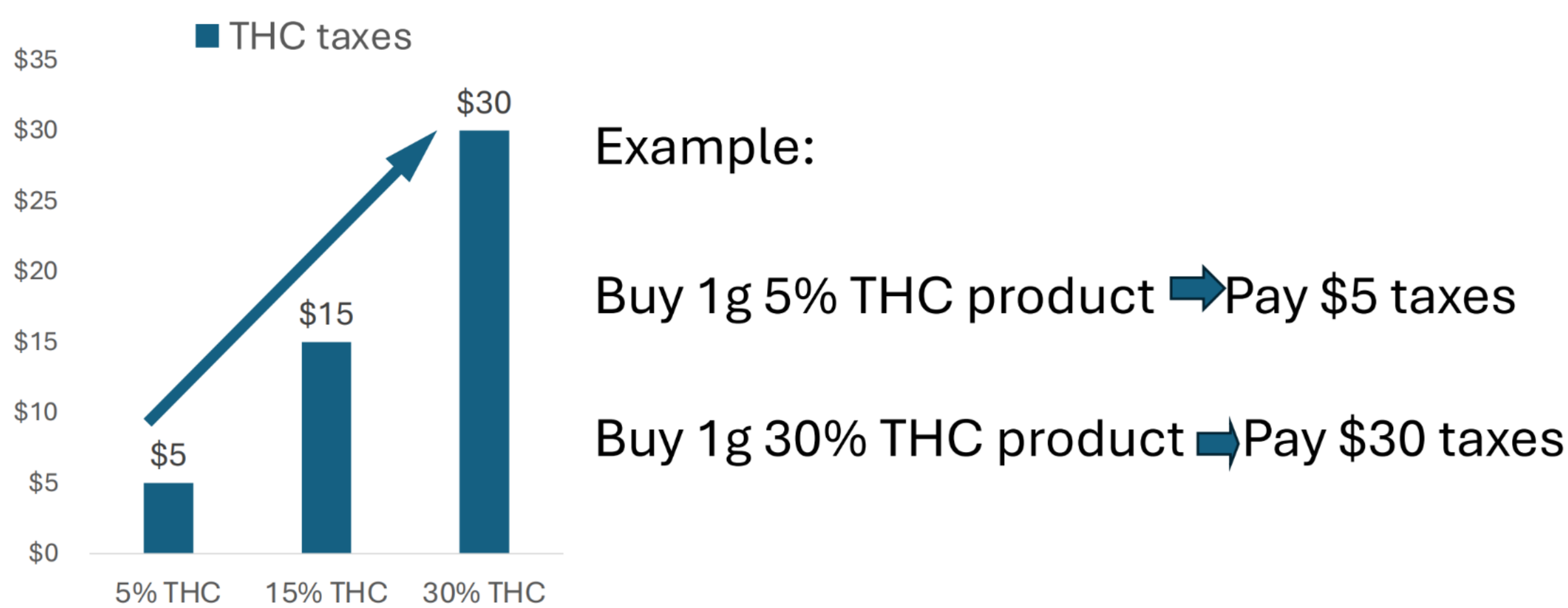

Notes: The figure illustrates the design of the THC-based tax condition, where taxes increase proportionally with product potency (e.g., $5 for 5% THC, $15 for 15% THC, and

$30 for 30% THC). The example on the right shows that a 1-gram product with 5% THC incurs a $5 tax, while a 30% THC product incurs a $30 tax.

Figure B.3 Example of Price-Based Cannabis Tax Stimuli

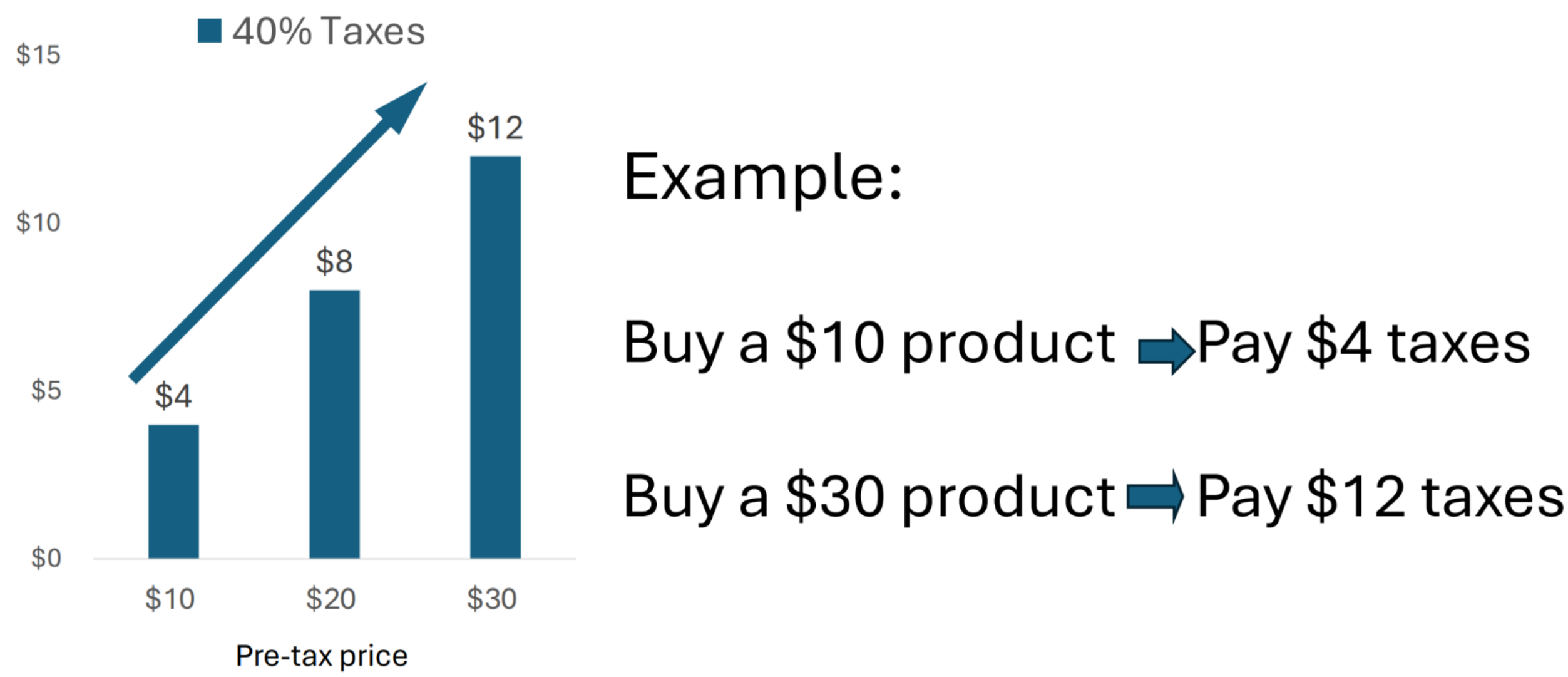

Notes: The figure illustrates the design of the price-based tax condition, where taxes are calculated as a percentage of the pre-tax price (e.g., a 40% tax results in $4 for a $10 product and $12 for a $30 product). This example shows how total taxes increase proportionally with the product's pre-tax price level.

Figure B.4 Example of the Cannabis Purchase Scenario Interface in the Experimental Task.

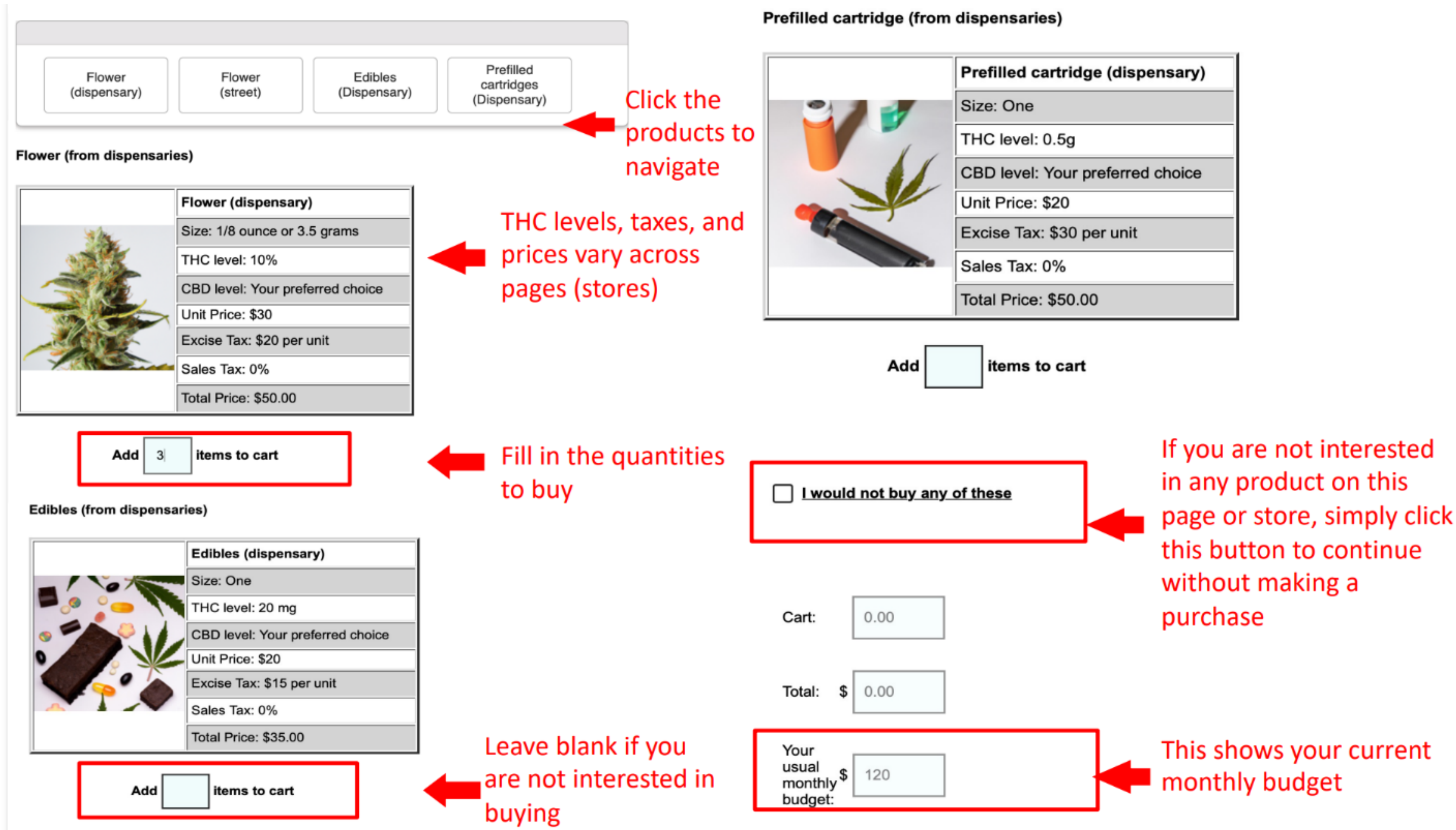

Notes: Participants navigated across product tabs (e.g., flower, edibles, prefilled cartridges) to view THC levels, prices, and taxes that varied across pages. They could specify the number of items to purchase, leave fields blank if not interested, or select "I would not buy any of these" to skip the choice set. The bottom of the interface displayed the participant's usual monthly budget to guide purchase decisions.
