# Supplementary material for: The Impact of Various Tax Bases and Rates on Cannabis Consumption Among US Adults Who use Recreational Cannabis
Source: Health Econ. 2026 Jul 8;35(10):1519–31. doi: 10.1002/hec.70129 (PMC13508039; doi:10.1002/hec.70129)
Supplement: Supplementary file 2 [file HEC-35-1519-s001.docx]

**Choice set example/display with budget reminder**

Shop for products to use for the next month

Flower

(dispensary)

Flower

(street)

Edibles

(dispensary)

Cartridges

(dispensary)

**Flower (from dispensaries)**

|  | **Flower (dispensary)** |
| --- | --- |
|  | Size: 1/8 ounce or 3.5 grams |
|  | THC level: XX% or XX mg |
|  | CBD level: Your preferred choice |
|  | Unit Price: $XX |
|  | Excise Tax: [Insert rate & base] |
|  | Sales Tax: $XX |
|  | Total Price: $XX |

**Add [	] items to cart**

**Flower (from street vendors or friends)**

|  | **Flower (street vendor)** |
| --- | --- |
|  | Size: 1/8 ounce or 3.5 grams |
|  | THC level: XX% or XX mg |
|  | CBD level: Your preferred choice |
|  | Unit Price: $XX |
|  | Excise Tax: None |
|  | Sales Tax: None |
|  | Total Price: : $XX |

**Add [	] items to cart**

**Edibles (from dispensaries)**

|  | **Edibles (dispensary)** |
| --- | --- |
|  | Size: One |
|  | THC level: XX% or XX mg |
|  | CBD level: Your preferred choice |
|  | Unit Price: $XX |
|  | Excise Tax: [Insert rate & base] |
|  | Sales Tax: $XX |
|  | Total Price: $XX |

**Add [	] items to cart**

**Prefilled cartridge (from dispensaries)**

|  | **Prefilled cartridge (dispensary)** |
| --- | --- |
|  | Size: One |
|  | THC level: XX% or XX mg |
|  | CBD level: Your preferred choice |
|  | Unit Price: $XX |
|  | Excise Tax: [Insert rate & base] |
|  | Sales Tax: $XX |
|  | Total Price: $XX |

**Add [	] items to cart**

**I would not buy any of these** [check box]

**Cart**: [fill in unit_total]

**Total**: $[fill in total_cost]

**Your usual monthly budget**: $[fill in monbudget]

<< Store 1 out of # >>

[continue to stores 2-#]
