# Supplementary material for: The Impact of Various Tax Bases and Rates on Cannabis Consumption Among US Adults Who use Recreational Cannabis
Source: Health Econ. 2026 Jul 8;35(10):1519–31. doi: 10.1002/hec.70129 (PMC13508039; doi:10.1002/hec.70129)
Supplement: Supplementary file 3 [file HEC-35-1519-s003.docx]

**Instruction with Cheap talk script embedded**

Imagine you’re low on your supply, and it is time to stock up on some cannabis products for the **next month**. You’ve checked out **9** different online stores that sell all sorts of cannabis stuff—like flower, edibles, and those prefilled cartridges.

Now, here’s the deal: each store has its own mix of **potency levels** (that’s the THC content) and **prices** (including taxes). So, we’re curious: **Which products would you pick and how many would you buy?** It’s like your regular cannabis shopping experience. Share your picks!

[**page break]**

Picture this: you are cruising through the store webpages, checking out all the products. There is a real-time tracker that shows you how many items you have added to your cart and how much it all costs.

And there is more. Your monthly budget for cannabis, which is set based on your answers to some budgeting questions earlier, is right there to help you make choices. We want you to stick to that budget, just like you would in real-world shopping. However, if you need to go a bit beyond your budget, that will be OK too. Just remember to shop as you would in real world.

Here's how the task looks like if you had a monthly budget of $120:

**[Insert Image cannabis[7]]**

If you are taking this task on a device with a small screen, you may need to scroll down and to the right in order to see all the options.

[**page break]**

Once you feel ready, you can proceed to the next page to start the task.
