# Supplementary material for: The Impact of Various Tax Bases and Rates on Cannabis Consumption Among US Adults Who use Recreational Cannabis
Source: Health Econ. 2026 Jul 8;35(10):1519–31. doi: 10.1002/hec.70129 (PMC13508039; doi:10.1002/hec.70129)
Supplement: Supplementary file 4 [file HEC-35-1519-s004.docx]

Appendix A

**Figure A.1 The THC Unit by Product Category**

*Notes:* In Figure A.1, for all categories, the average THC unit is 668, with a standard deviation of 3,935.65; 75.48% observations have zero THC unit. For legal flower consumption, the average THC unit is 740 (SD: 4,229.39); For illegal flower consumption, the average THC unit is 1649.20 (SD: 6,306.49); For Edible, the average THC unit is 104.97 (SD: 510.63); For Concentrate, the average THC unit is 177.37 (SD: 1,585.74).

**Figure A.2 The Unit by Product Category**

*Notes:* In Figure A.2, for all categories, the average unit is 1.02, with a standard deviation of 5.30; 75.48% observations have zero units. For legal flower consumption, the average unit is 0.96 (SD: 4.96); For illegal flower consumption, the average unit is 2.22 (SD: 8.67); For edible, the average unit is 0.64 (SD: 2.24); For Concentrate, the average unit is 0.24 (SD: 2.29).

**Table A.1: Summary Statistics (Observation = 1,525)**

| VARIABLES | Percent/Mean (SD) |
| --- | --- |
| Sociodemographic Factors | |
| Age | 48.80 (15.30) |
| Gender |  |
| Female | 45.70% |
| Male | 53.97% |
| Do not know | 0.33% |
| Race/Ethnicity |  |
| White, non-Hispanic | 72.66% |
| Black, non-Hispanic | 11.02% |
| Other, non-Hispanic | 1.44% |
| Hispanic | 10.75% |
| 2+ Races, non-Hispanic | 4.13% |
| Education |  |
| No High School or GED | 5.77% |
| High School Graduate | 20.33% |
| Some College | 32.26% |
| Bachelor’s Degree or Higher | 41.64% |
| Household Income |  |
| Less than $10,000 | 4.59% |
| $10,000 to $24,999 | 11.41% |
| $25,000 to $49,999 | 17.51% |
| $50,000 to $74,999 | 14.95% |
| $75,000 to $99,999 | 14.03% |
| $100,000 to $149,999 | 16.72% |
| $150,000 or more | 20.79% |
| Cannabis use and spending |  |
| Monthly Cannabis Expenditure | 118.41 (172.35) |
| % expenditure spent by forms |  |
| Flowers | 46.87% |
| Edibles | 27.76% |
| Cartridges | 13.21% |
| Others | 12.16% |
| Daily use | 54.07% |
| All purchases are from legal sources | 54.30% |

**Table A.2: Other Factors Contributing to Cannabis Consumption, Estimated Using**

**Zero-Inflated Negative Binomial Regressions, N=53,480**

|  | | (1) | | (2) | | (3) | | | (4) |
| --- | --- | --- | --- | --- | --- | --- | --- | --- | --- |
| VARIABLES | | Outcome = Unit | | | | Outcome = THC Unit | | | |
| Within-Subject Effects: | | | | |  | | | | |
| Product Constant (Base: Legal Flower) | | | | | | | | | |
| Illegal Flower | | 0.544^***^ | | 0.588^***^ | | 0.576^***^ | | | 0.609^***^ |
|  | | (0.080) | | (0.064) | | (0.075) | | | (0.059) |
| Edible | | -0.130 | | -0.046 | | -2.030^***^ | | | -1.940^***^ |
|  | | (0.105) | | (0.078) | | (0.099) | | | (0.074) |
| Cartridge | | -1.452^***^ | | -1.404^***^ | | -1.569^***^ | | | -1.522^***^ |
|  | | (0.108) | | (0.088) | | (0.102) | | | (0.084) |
| Between-Subject Effects: | | |  | | | |  | | |
| Expenditures | | 0.430*** | | 0.448*** | | 0.373*** | | | 0.386*** |
|  | | (0.027) | | (0.027) | | (0.033) | | | (0.034) |
| Age | | -0.037 | | -0.073 | | 0.074 | | | 0.047 |
|  | | (0.130) | | (0.099) | | (0.103) | | | (0.077) |
| Gender (Base: Male) |  | | | |  | | |  | |
| Female | | 0.118 | | 0.129^*^ | | 0.103 | | | 0.112^*^ |
|  | | (0.075) | | (0.054) | | (0.061) | | | (0.045) |
| Not Reported | | 0.331 | | 0.340 | | 0.006 | | | -0.028 |
|  | | (0.325) | | (0.345) | | (0.217) | | | (0.238) |
| Race and Ethnicity (Base: White, Non-Hispanic) | | | | | | |  | | |
| Black, non-Hispanic | | 0.323^*^ | | 0.265^*^ | | 0.076 | | | 0.062 |
|  | | (0.130) | | (0.111) | | (0.099) | | | (0.082) |
| Other, non-Hispanic | | -0.061 | | -0.033 | | -0.012 | | | 0.003 |
|  | | (0.159) | | (0.128) | | (0.149) | | | (0.131) |
| Hispanic | | 0.225 | | 0.143 | | 0.093 | | | 0.007 |
|  | | (0.143) | | (0.095) | | (0.123) | | | (0.083) |
| 2+ Races, non-Hispanic | | -0.058 | | -0.058 | | 0.104 | | | 0.124 |
|  | | (0.118) | | (0.121) | | (0.116) | | | (0.116) |
| Household Income (Base: Less than $10,000) | | | | | |  | | |  |
| $10,000 to $24,999 | | -0.440 | | -0.401 | | -0.239 | | | -0.213 |
|  | | (0.291) | | (0.218) | | (0.209) | | | (0.170) |
| $25,000 to $49,999 | | -0.501 | | -0.444^*^ | | -0.298 | | | -0.254 |
|  | | (0.260) | | (0.185) | | (0.179) | | | (0.146) |
| $50,000 to $74,999 | | -0.232 | | -0.285 | | -0.101 | | | -0.188 |
|  | | (0.294) | | (0.209) | | (0.215) | | | (0.153) |
| $75,000 to $99,999 | | -0.301 | | -0.337 | | -0.165 | | | -0.192 |
|  | | (0.270) | | (0.191) | | (0.192) | | | (0.145) |
| $100,000 to $149,999 | | -0.236 | | -0.194 | | -0.153 | | | -0.124 |
|  | | (0.273) | | (0.202) | | (0.187) | | | (0.152) |
| $150,000 or more | | -0.359 | | -0.273 | | -0.313 | | | -0.224 |
|  | | (0.263) | | (0.197) | | (0.178) | | | (0.146) |
| Education (Base: No High School Diploma or GED) | | | | | |  | | |  |
| High School Graduate | | -0.067 | | -0.178 | | 0.093 | | | -0.036 |
|  | | (0.183) | | (0.146) | | (0.159) | | | (0.130) |
| Some College | | -0.452^**^ | | -0.420^***^ | | -0.342^**^ | | | -0.341^***^ |
|  | | (0.141) | | (0.124) | | (0.110) | | | (0.102) |
| Bachelor's or higher | | -0.402^**^ | | -0.400^**^ | | -0.321^**^ | | | -0.345^**^ |
|  | | (0.156) | | (0.135) | | (0.121) | | | (0.108) |
| State Fixed Effects | | No | | Yes | | No | | | Yes |

Note: Semi-elasticities are reported for categorical variables. Standard errors clustered at the individual level are in parentheses ^*^ *p* < 0.05, ^**^ *p* < 0.01, ^***^ *p* < 0.001

**Table A.3: Own- and Cross-Price Elasticities of Demand Among Different**

**Cannabis Forms, Estimated using Zero-Inflated Negative Binomial Regressions, N=53,480**

|  | | (1) | | (2) | | (3) | | | (4) |
| --- | --- | --- | --- | --- | --- | --- | --- | --- | --- |
| VARIABLES | | Outcome = Unit | | | | Outcome = THC Unit | | | |
| Within-Subject Effects: | | | | |  | | | | |
| Product Constant (Base: Legal Flower) | | | | | | | | | |
| Illegal Flower | | 0.697^*^ | | 0.590^*^ | | 0.269 | | | 0.297 |
|  | | (0.304) | | (0.240) | | (0.220) | | | (0.205) |
| Edible | | -0.425 | | -0.481 | | -1.298^***^ | | | -1.101^***^ |
|  | | (1.157) | | (0.293) | | (0.267) | | | (0.235) |
| Cartridge | | -0.772 | | -0.194 | | -0.802^**^ | | | -0.908^***^ |
|  | | (0.633) | | (0.422) | | (0.304) | | | (0.253) |
| THC Levels (Base: 10%) | |  | |  | |  | | |  |
| 15% | | 0.166^**^ | | 0.110 | | 0.738^***^ | | | 0.716^***^ |
|  | | (0.052) | | (0.061) | | (0.059) | | | (0.047) |
| 25% | | 0.246^***^ | | 0.210^***^ | | 1.320^***^ | | | 1.323^***^ |
|  | | (0.048) | | (0.053) | | (0.050) | | | (0.041) |
| 30% | | 0.386^***^ | | 0.344^***^ | | 2.092^***^ | | | 2.076^***^ |
|  | | (0.053) | | (0.055) | | (0.058) | | | (0.048) |
| Between-Subject Effects: | | |  | | | |  | | |
| Bases of Tax Imposed (Base: Weight) | | | | | | |  | | |
| Price | | 0.106 | | 0.124 | | 0.168^*^ | | | 0.098 |
|  | | (0.090) | | (0.087) | | (0.085) | | | (0.065) |
| THC | | -0.006 | | 0.081 | | 0.054 | | | 0.021 |
|  | | (0.088) | | (0.094) | | (0.083) | | | (0.071) |
| Expenditures | | 0.430*** | | 0.303^***^ | | 0.373*** | | | 0.386*** |
|  | | (0.027) | | (0.022) | | (0.033) | | | (0.034) |
| Age | | -0.050 | | 0.074 | | 0.061 | | | 0.046 |
|  | | (0.125) | | (0.146) | | (0.099) | | | (0.077) |
| Gender (Base: Male) |  | | | |  | | |  | |
| Female | | 0.118 | | 0.129^*^ | | 0.103 | | | 0.112^*^ |
|  | | (0.075) | | (0.054) | | (0.061) | | | (0.045) |
| Not Reported | | 0.331 | | 0.340 | | 0.006 | | | -0.028 |
|  | | (0.325) | | (0.345) | | (0.217) | | | (0.238) |
| Race and Ethnicity (Base: White, Non-Hispanic) | | | | | | |  | | |
| Black, non-Hispanic | | 0.323^*^ | | 0.021 | | 0.084 | | | 0.065 |
|  | | (0.126) | | (0.153) | | (0.097) | | | (0.081) |
| Other, non-Hispanic | | -0.027 | | 0.085 | | 0.016 | | | 0.018 |
|  | | (0.159) | | (0.221) | | (0.144) | | | (0.128) |
| Hispanic | | 0.239 | | -0.068 | | 0.101 | | | 0.005 |
|  | | (0.139) | | (0.221) | | (0.122) | | | (0.083) |
| 2+ Races, non-Hispanic | | -0.039 | | 0.171 | | 0.128 | | | 0.147 |
|  | | (0.119) | | (0.202) | | (0.119) | | | (0.119) |
| Household Income (Base: Less than $10,000) | | | | | |  | | |  |
| $10,000 to $24,999 | | -0.428 | | -0.262 | | -0.221 | | | -0.190 |
|  | | (0.278) | | (0.264) | | (0.203) | | | (0.169) |
| $25,000 to $49,999 | | -0.488 | | -0.326 | | -0.280 | | | -0.239 |
|  | | (0.251) | | (0.244) | | (0.176) | | | (0.146) |
| $50,000 to $74,999 | | -0.218 | | -0.073 | | -0.080 | | | -0.168 |
|  | | (0.283) | | (0.270) | | (0.211) | | | (0.153) |
| $75,000 to $99,999 | | -0.298 | | -0.126 | | -0.155 | | | -0.180 |
|  | | (0.257) | | (0.236) | | (0.186) | | | (0.145) |
| $100,000 to $149,999 | | -0.227 | | -0.214 | | -0.144 | | | -0.116 |
|  | | (0.263) | | (0.240) | | (0.183) | | | (0.151) |
| $150,000 or more | | -0.343 | | -0.225 | | -0.295 | | | -0.204 |
|  | | (0.253) | | (0.230) | | (0.176) | | | (0.146) |
| Education (Base: No High School Diploma or GED) | | | | | |  | | |  |
| High School Graduate | | -0.075 | | 0.066 | | 0.121 | | | -0.012 |
|  | | (0.176) | | (0.192) | | (0.156) | | | (0.130) |
| Some College | | -0.450^**^ | | -0.253 | | -0.315^**^ | | | -0.316^**^ |
|  | | (0.138) | | (0.141) | | (0.108) | | | (0.103) |
| Bachelor or higher | | -0.414^**^ | | -0.289 | | -0.301^*^ | | | -0.322^**^ |
|  | | (0.152) | | (0.159) | | (0.119) | | | (0.108) |
| State Fixed Effects | | No | | Yes | | No | | | Yes |

Standard errors in parentheses

^*^ *p* < 0.05, ^**^ *p* < 0.01, ^***^ *p* < 0.001

| **Table A.4: Subgroup Analyses of the Impacts of Attributes on THC Consumption, Estimated Using ZINB Regressions** | | | | | | | | |
| --- | --- | --- | --- | --- | --- | --- | --- | --- |
|  | (1) | | (2) | | (3) | | | (4) |
| GROUP | Daily | | Nondaily | | 21-40 | | | >40 |
| N | 28,724 | | 24,684 | | 18,960 | | | 34,520 |
| Within-Subject Effects: | |  | | | |  | | |
| Pre-Tax Price Levels (Base: $10 for flowers and edibles; $ 20 for cartridges) | | | | | | | | |
| 100% increase | -0.538^***^ | | -0.551^***^ | | -0.590^***^ | | | -0.503^***^ |
|  | (0.038) | | (0.034) | | (0.043) | | | (0.032) |
| 200% increase | -0.955^***^ | | -0.906^***^ | | -1.043^***^ | | | -0.851^***^ |
|  | (0.046) | | (0.047) | | (0.054) | | | (0.041) |
| 300% increase | -1.241^***^ | | -1.179^***^ | | -1.400^***^ | | | -1.094^***^ |
|  | (0.065) | | (0.059) | | (0.072) | | | (0.057) |
| THC Levels (Base: low) | | | | | | | | |
| Medium low | 0.734^***^ | | 0.744^***^ | | 0.745^***^ | | | 0.701^***^ |
|  | (0.060) | | (0.059) | | (0.069) | | | (0.055) |
| Medium high | 1.385^***^ | | 1.298^***^ | | 1.278^***^ | | | 1.375^***^ |
|  | (0.054) | | (0.055) | | (0.060) | | | (0.054) |
| High | 1.892^***^ | | 2.121^***^ | | 1.947^***^ | | | 1.978^***^ |
|  | (0.058) | | (0.062) | | (0.069) | | | (0.055) |
| Tax Levels (Base: 20% or equivalent) | | | | | | | | |
| 40% or equivalent | -0.194^**^ | | -0.231^***^ | | -0.265^***^ | | | -0.157^**^ |
|  | (0.061) | | (0.064) | | (0.062) | | | (0.059) |
| 60% or equivalent | -0.286^***^ | | -0.348^***^ | | -0.277^***^ | | | -0.343^***^ |
|  | (0.048) | | (0.042) | | (0.053) | | | (0.040) |
| 80% or equivalent | -0.351^***^ | | -0.407^***^ | | -0.402^***^ | | | -0.338^***^ |
|  | (0.050) | | (0.053) | | (0.062) | | | (0.043) |
| Between-Subject Effects: | | | |  | | |  | |
| Bases of Tax Imposed (Base: Weight) | | | | | | |  | |
| Price | 0.123 | | 0.045 | | 0.112 | | | 0.154 |
|  | (0.080) | | (0.098) | | (0.097) | | | (0.080) |
| THC | 0.070 | | -0.099 | | 0.109 | | | 0.017 |
|  | (0.086) | | (0.103) | | (0.100) | | | (0.088) |

Notes: State fixed effects and other covariates are controlled for. Marginal effects or semi-elasticities estimated using eydx are reported. Standard errors clustered at the individual levels are in parentheses. ^*^ *p* < 0.05, ^**^ *p* < 0.01, ^***^ *p* < 0.001.

**Table A.5: Economic Relationship Based on THC Consumption between Different Forms in the Matrix.**

| Price  Consumption | Legal flowers | Illegal flowers | Edibles | Cartridges |
| --- | --- | --- | --- | --- |
| Legal flowers | Reduction | Substitutes |  |  |
|  |  |  |  |  |
| Illegal flowers | Substitutes | Reduction |  | Substitutes |
| Edibles | Complements | Complements | Reduction | Complements |
|  |  |  |  |  |
| Cartridges |  | Complements |  | Reduction |
|  |  |  |  |  |

Matrix of relationships based on Table 3, Column 4.
